# Assessment of the genomic prediction accuracy for feed efficiency traits in meat-type chickens

**DOI:** 10.1371/journal.pone.0173620

**Published:** 2017-03-09

**Authors:** Tianfei Liu, Chenglong Luo, Jie Wang, Jie Ma, Dingming Shu, Mogens Sandø Lund, Guosheng Su, Hao Qu

**Affiliations:** 1 Institute of Animal Science, Guangdong Academy of Agricultural Sciences, Guangzhou, China; 2 State Key Laboratory of Livestock and Poultry Breeding, Guangzhou, China; 3 Guangdong Key Laboratory of Animal Breeding and Nutrition, Guangzhou, China; 4 Center for Quantitative Genetics and Genomics, Department of Molecular Biology and Genetics, Aarhus University, Denmark; China Agricultural University, CHINA

## Abstract

Feed represents the major cost of chicken production. Selection for improving feed utilization is a feasible way to reduce feed cost and greenhouse gas emissions. The objectives of this study were to investigate the efficiency of genomic prediction for feed conversion ratio (FCR), residual feed intake (RFI), average daily gain (ADG) and average daily feed intake (ADFI) and to assess the impact of selection for feed efficiency traits FCR and RFI on eviscerating percentage (EP), breast muscle percentage (BMP) and leg muscle percentage (LMP) in meat-type chickens. Genomic prediction was assessed using a 4-fold cross-validation for two validation scenarios. The first scenario was a random family sampling validation (CVF), and the second scenario was a random individual sampling validation (CVR). Variance components were estimated based on the genomic relationship built with single nucleotide polymorphism markers. Genomic estimated breeding values (GEBV) were predicted using a genomic best linear unbiased prediction model. The accuracies of GEBV were evaluated in two ways: the correlation between GEBV and corrected phenotypic value divided by the square root of heritability, i.e., the correlation-based accuracy, and model-based theoretical accuracy. Breeding values were also predicted using a conventional pedigree-based best linear unbiased prediction model in order to compare accuracies of genomic and conventional predictions. The heritability estimates of FCR and RFI were 0.29 and 0.50, respectively. The heritability estimates of ADG, ADFI, EP, BMP and LMP ranged from 0.34 to 0.53. In the CVF scenario, the correlation-based accuracy and the theoretical accuracy of genomic prediction for FCR were slightly higher than those for RFI. The correlation-based accuracies for FCR, RFI, ADG and ADFI were 0.360, 0.284, 0.574 and 0.520, respectively, and the model-based theoretical accuracies were 0.420, 0.414, 0.401 and 0.382, respectively. In the CVR scenario, the correlation-based accuracy and the theoretical accuracy of genomic prediction for FCR was lower than RFI, which was different from the CVF scenario. The correlation-based accuracies for FCR, RFI, ADG and ADFI were 0.449, 0.593, 0.581 and 0.627, respectively, and the model-based theoretical accuracies were 0.577, 0.629, 0.631 and 0.638, respectively. The accuracies of genomic predictions were 0.371 and 0.322 higher than the conventional pedigree-based predictions for the CVF and CVR scenarios, respectively. The genetic correlations of FCR with EP, BMP and LMP were -0.427, -0.156 and -0.338, respectively. The correlations between RFI and the three carcass traits were -0.320, -0.404 and -0.353, respectively. These results indicate that RFI and FCR have a moderate accuracy of genomic prediction. Improving RFI and FCR could be favourable for EP, BMP and LMP. Compared with FCR, which can be improved by selection for ADG in typical meat-type chicken breeding programs, selection for RFI could lead to extra improvement in feed efficiency.

## Introduction

Feed represents up to 70 percent of the total cost of chicken production, and the selection of feed utilization traits can reduce feed cost and greenhouse gas emissions[1]. Feed conversion ratio (FCR) and residual feed intake (RFI) are widely used to measure feed efficiency in animal production [2–5]. Both FCR and RFI have moderate heritability; consequently, genetic selection for any of the two traits can improve feed efficiency in chickens [2, 6–8]. FCR is defined as the ratio of feed intake to body weight gain. As a ratio of two traits, FCR is not normally distributed and there is a high correlation between the two component traits. This makes it difficult to predict the real response to selection for this trait [9]. RFI is defined as the difference between actual and expected feed intake given body weight and weight gain [10]. Because RFI is independent of body weight and weight gain, selection for RFI can improve feed efficiency without changing the production traits [2].

Genomic selection is revolutionizing animal breeding due to the higher accuracy of selection early in life [11–13]. Genomic selection selects breeding animals based on breeding values that are estimated using genomic marker information [14]. This selection method benefits the traits that are difficult and expensive to measure, such as feed efficiency traits [15]. Several studies have demonstrated that genomic selection is superior to the conventional method, which is based on pedigree information in poultry [7, 16, 17]. In laying hens, Wolc et al. [7] reported that the accuracy from genomic prediction for feed utilization traits was higher and more persistent than from conventional selection and suggested using genomic selection to improve feed efficiency.

The aims of this study were to investigate the accuracy of genomic prediction for FCR and RFI and to assess the impact of improving the two feed efficiency traits on carcass traits in meat-type chickens.

## Materials and methods

### Ethics approval

The present study was approved by the Animal Care Committee of the Institute of Animal Science, Guangdong Academy of Agricultural Sciences (Guangzhou, People's Republic of China) (Approval No. GAAS-IAS-2009-73).

### Population and data

The chicken population was constructed by crossing the “High Quality chicken Line A” (HQLA) with the Huiyang Beard chicken (HB), as described in Sheng et al. [18]. The HQLA line is a fast-growing Chinese yellow broiler line, which has been under selection for growth traits and high meat quality tailored to Chinese tastes for more than 10 generations. The HB line is a Chinese indigenous breed, which is characterized by slow growth and high meat quality. The data included 582 birds from three generations with the structure of 20 breeding individuals (6♂ + 14♀) from the F_0_ generation, 51 breeding individuals (8♂ + 43♀) from the F_1_ generation and 511 individuals from eight half-sib families of the F_2_ generation. All birds were genotyped, and only F_2_ birds had phenotypic records. The birds were genotyped using the Illumina Chicken 60K SNP Beadchip [19] by DNA LandMarks Inc., SaintJean-sur-Richelieu, Canada. The SNP marker data were screened using the criteria of call rate per SNP marker greater than 95%, a GenTrain score greater than 0.6, and minor allele frequency greater than 0.01. Because the population originated from a cross between two selected lines, the dataset did not have a check for Hardy-Weinberg equilibrium. After editing, there were 46,672 SNPs in the marker data.

Seven traits in the analysis included feed conversion ratio (FCR), residual feed intake (RFI), average daily gain (ADG), average daily feed intake (ADFI), eviscerating percentage (EP), breast muscle percentage (BMP) and leg muscle percentage (LMP). The mean and standard deviation of the seven traits are shown in Table 1. The chicks were kept in group cages and fed a starter ration containing 2,900 kcal ME/kg and 200 g/kg CP until the end of 5^th^ week. Thereafter, all birds were housed in single cages and feed was provided in single containers. The chickens had *ad libitum* access to water and feed. The feed were gradually changed to a grower ration of 2,950 kcal ME/kg and 180 g/kg CP during the 6^th^ week. Body weight and feed intake were measured during the period from the beginning of 7^th^ to the end of 12^th^ week (42 d). Individual body weight gain was calculated based on body weight between the start and end of the period and used to derive ADG. The total feed intake for each bird was calculated by summing the feed consumptions during the period and used to derive ADFI. At the end of the 13^th^ week, the birds were slaughtered to measure carcass traits. Quality control was applied to ADG and ADFI, removing deviant data that were more than 3 standard deviations from mean [20]. Finally, 7 birds were excluded from the analysis, and the remaining 504 records were available for the analysis. FCR was calculated as the ratio of ADFI to ADG. RFI was defined as the difference between observed ADFI and expected ADFI, obtained from an analysis using the model [21]:

$$ADFI=\mu +sex+hatch+\beta_{1}{MBW}^{0.75}+\beta_{2}ADG+e.$$

In the model, *μ* is the intercept, *sex* and *hatch* are fixed effects, *MBW* is the average of the start and end of body weight, *β*_*1*_ and *β*_*2*_ are partial regression coefficients, and *e* is residual. The estimated *e* was taken as the measure of RFI.

**Table 1:** Mean and standard deviation of the traits.

Trait^1^	N	Mean	SD
**FCR**	504	3.54	0.37
**RFI**	504	0.00	4.62
**ADG**	504	29.34	5.82
**ADFI**	504	102.36	14.41
**EP**	504	66.60	1.81
**BMP**	504	17.40	1.44
**LMP**	504	23.72	2.58

^1^ FCR = feed conversion ratio, RFI = residual feed intake, ADG = average daily gain (g), ADFI = average daily feed intake (g), EP = eviscerating percentage (%), BMP = breast muscle percentage (%), LMP = leg muscle percentage (%).

### Statistical model

In this study, a conventional pedigree-based best linear unbiased prediction model (BLUP) and a genomic best linear unbiased prediction model (GBLUP) were used to predict breeding values for each trait, respectively.

The pedigree-based BLUP model[22] is

y=Xb+Za+e,

where **y** is the vector of phenotypic records of the trait (FCR, RFI, ADG or ADFI), **b** is the vector of the fixed effect (sex and hatch), **X** is the incidence matrix linking **b** to **y**, **a** is a vector of additive breeding values to be estimated, **Z** is the incidence matrices linking **a** to **y** and **e** is the vector of residuals. It was assumed that $a∼N(0,A\sigma_{a}^{2})$, where **A** is the pedigree-based genetic relationship matrix. For analysis of RFI, the fixed effects were not included in the model because these effects were already corrected when calculating RFI values.

The GBLUP model [23] is

y=Xb+Zg+e,

where the definitions of **y**, **b**, **X**, **Z** and **e** are the same as those in the BLUP model. **g** is a vector of genomic breeding values to be estimated. It was assumed that **g** ∼ **N**(**0**,**G**⊗**V**_**g**_), where **G** is the genomic relationship matrix based on SNP markers [23], **V**_**g**_ is the covariance matrix of genomic breeding values.

Variance components were estimated with a univariate linear mixed model, the genetic correlations between feed efficiency and each of the carcass traits were estimated with a multivariate linear mixed model, using the average information restricted maximum likelihood (AIREML) approach [24]. The linear mixed models were exactly consistent with the GBLUP models, and the whole data were used to estimate variance and covariance components. The estimation of variance components and the prediction of breeding values were carried out using the DMU package [25].

### Cross-validation

Cross-validation is a robust, non-parametric technique for assessment of predictive ability [11, 26]. The advantage of multiple-fold cross validation is that it can retain both the training data and the total test data as large as possible, which is very useful especially when the whole data set is small. In this study, a 4-fold cross-validation was applied to assess the accuracy of genomic predictions. Two scenarios of validation were performed. The details of sampling methods to create subsets were described in the previous study [12]. Briefly, in the first cross-validation scenario, the eight half-sib families were randomly divided into four subsets (family sampling, CVF), each including two families. In the second scenario, 504 birds were randomly divided into four subsets (individual sampling, CVR), each including 126 birds. In each fold validation, one subset was used as test data and the other subsets as training data. Thus, in CVF, the test birds did not have any sibs in the corresponding training data, indicating a relatively distant relationship between test and training birds. Conversely, in CVR, the test birds had sibs in training birds, indicating a relatively close relationship between test and training birds. To account for population structure and sampling variation, splitting was repeated 10 times in CVF and 50 times in CVR. In CVF scenarios, because the numbers of individuals in each family were not equal, the numbers in the training and test data sets varied from 283 to 481 and from 23 to 221, respectively. In CVR scenarios, each fold had the same number of birds, i.e., the numbers of birds in the test and training data sets were 378 and 126, respectively.

Accuracies of GEBV were assessed in two ways. One was the correlation between prediction and corrected phenotypic value (y_c_) divided by the square root of heritability [27], where y_c_ was the phenotypic value corrected for sex and batch effects. For RFI, the y_c_ was RFI itself, as RFI was already corrected for sex and batch effects. The other was the model-based theoretical accuracy of GEBV obtained by inverting the coefficient matrix of the mixed model equations [22].

## Results

Estimates of variance components and heritability of FCR, RFI, ADG, ADFI, EP, BMP and LMP are presented in Table 2. FCR and RFI had moderate heritability of 0.29 and 0.50, respectively. ADG, ADFI, EP, BMP and LMP also had moderate heritability ranged from 0.34 to 0.53. Despite the small data set, the standard error of estimated heritability was small and ranged between 0.07 and 0.08.

**Table 2:** Genetic parameters estimated from all data.

**Trait**^1^	$\sigma_{a}^{2}$	$\sigma_{e}^{2}$	$h_{a}^{2}$
**FCR**	0.02	0.06	0.29±0.08
**RFI**	11.06	11.23	0.50±0.07
**ADG**	8.74	9.14	0.49±0.07
**ADFI**	73.33	65.96	0.53±0.07
**EP**	1.06	1.68	0.39±0.07
**BMP**	0.67	1.30	0.34±0.08
**LMP**	1.17	1.07	0.52±0.08

^1^ FCR = feed conversion ratio, RFI = residual feed intake, ADG = average daily gain, ADFI = average daily feed intake, EP = eviscerating percentage, BMP = breast muscle percentage, LMP = leg muscle percentage.

The accuracy of genomic predictions in the test sets of the cross-validation are shown in Table 3, and the details of the correlations are shown in S1 Table as an additional file. Two factors reflected the accuracies of genomic predictions in CVF and CVR scenarios. One was the correlation-based accuracy, and the other was model-based theoretical accuracy. In the CVF scenario, the correlation-based accuracy and the theoretical accuracy of genomic prediction for FCR were slightly higher than those for RFI. The correlation-based accuracies for FCR, RFI, ADG and ADFI were 0.360, 0.284, 0.574 and 0.520, respectively. The theoretical accuracies of predictions for FCR, RFI, ADG and ADFI were 0.420, 0.414, 0.401 and 0.382, respectively. In the CVR scenario, the correlation-based accuracy and the theoretical accuracy of genomic prediction for FCR were lower than RFI, which was different from the CVF scenario. The correlation-based accuracies of predictions for FCR, RFI, ADG and ADFI were 0.241, 0.417, 0.406 and 0.455, respectively. The theoretical accuracies of predictions for FCR, RFI, ADG and ADFI were 0.577, 0.629, 0.631 and 0.638, respectively. The accuracies of the conventional pedigree prediction ranged from -0.045 to 0.398. Clearly, the accuracies of genomic prediction were much higher than those of the conventional pedigree-based prediction for both the CVF and CVR scenarios.

**Table 3:** Accuracy of genomic predictions for the birds in the test sets of the cross-validation.

Trait^1^	r_cor___CVF_^2^	r_theo___CVF_	r_ped___CVF_	r_cor___CVR_	r_theo___CVR_	r_ped___CVR_
**FCR**	0.360	0.420	0.202	0.449	0.577	0.232
**RFI**	0.284	0.414	0.166	0.593	0.629	0.398
**ADG**	0.574	0.401	-0.045	0.581	0.631	0.099
**ADFI**	0.520	0.382	-0.068	0.627	0.638	0.236
**Mean**	**0.435**	**0.404**	**0.064**	**0.563**	**0.619**	**0.241**

^1^FCR = feed conversion ratio, RFI = residual feed intake, ADG = average daily gain, ADFI = average daily feed intake.

^2^r_cor_ = the correlation between genomic prediction and corrected phenotypic value divided by the square root of heritability, r_theo_ = model-based theoretical accuracy of genomic predictions calculated by inverting the coefficient matrix of the mixed model equations, r_ped_ = the correlation between pedigree prediction and corrected phenotypic value divided by the square root of heritability, CVF = the test sets of the cross-validation by random family sampling, CVR = the test sets of the cross-validation by random individual sampling.

Table 4 shows the performance of various traits with low or high FCR and RFI based on GEBV. The top 50 birds (efficient) selected based on RFI and FCR shared 31 common individuals, and the bottom 50 birds (inefficient) shared 22 common individuals. For FCR, the top 50 birds had higher body weight gain and feed intake than the bottom 50 individuals. The three carcass traits had higher values in the efficient group. For all three carcass traits, the differences between the two groups were significant (*P*<0.05). For RFI, the top 50 efficient birds were expected to have the same ADG as the bottom 50 inefficient birds because RFI was already adjusted for ADG. The small difference (not significant, *P*>0.05) was due to sampling error. The efficient individuals had lower feed intake than the inefficient individuals for RFI, which is not the same for FCR. The three carcass traits had significantly higher values in the efficient group of birds (*P*<0.05).

**Table 4:** Mean and standard deviation of the traits for the birds with high or low feed efficiency.

Trait^1^	ADG	ADFI	EP	BMP	LMP
**FCR_eff_**	31.75±5.58^a^	102.22±13.76^a^	67.66±1.24^a^	17.95±1.08^a^	24.26±2.27^a^
**FCR_ineff_**	25.6±5.22^b^	100.27±11.47^a^	65.92±1.85^b^	17.15±1.23^b^	22.91±3.08^b^
**RFI_eff_**	30.89±6.82^A^	99.31±16.08^B^	67.74±2.66^A^	18.07±1.44^A^	24.46±2.09^A^
**RFI_ineff_**	29.66±5.59^A^	109.42±12.64^A^	66.01±1.83^B^	16.69±1.40^B^	23.10±2.93^B^

^1^FCR_eff_ = the top 50 efficient chickens with low feed conversion ratio, FCR_ineff_ = the bottom 50 inefficient chickens with high feed conversion ratio, RFI_eff_ = the top 50 efficient chickens with low residual feed intake, RFI_ineff_ = the bottom 50 inefficient chickens with high residual feed intake, ADG = average daily gain (g), ADFI = average daily feed intake (g), EP = eviscerating percentage (%), BMP = breast muscle percentage (%), LMP = leg muscle percentage (%).

^a-b^ within a column for FCR, different superscripts indicate a significant difference (P<0.05), according to Student's t-test.

^A-B^ within a column for RFI, different superscripts indicate a significant difference (P<0.05), according to Student's t-test.

The genetic correlations between feed efficiency and carcass traits based on full data are presented in Table 5. Student's t-test was implemented to test the significance of the genetic correlation coefficients. The correlations between two feed efficiency traits and the three carcass traits were negative, and significantly differed from zero (*P*<0.05). The correlations of FCR with EP, BMP and LMP ranged from -0.427 to -0.156. The correlations between RFI and the three carcass traits ranged from -0.404 to -0.320.

**Table 5:** Genetic correlations between feed efficiency and carcass traits based on the whole data.

Trait^1^	EP	BMP	LMP
**FCR**	-0.427*	-0.156*	-0.338*
**RFI**	-0.320*	-0.404*	-0.353*

^1^FCR = feed conversion ratio, RFI = residual feed intake, EP = eviscerating percentage, BMP = breast muscle percentage, LMP = leg muscle percentage.

* Significantly different from 0 at P<0.05.

## Discussion

### Heritability of feed efficiency traits

Several studies have shown that the heritabilities for FCR and RFI are moderate to high in chickens [2, 6, 28, 29]. In Arkansas broilers, Aggrey et al. [2] reported that the heritability estimates of FCR and RFI were 0.41 and 0.42 at 5 to 6 weeks of age, respectively. Yuan et al. [6] studied the genetic parameters of FCR and RFI in two laying periods of hens and reported that the estimates of heritability for FCR and RFI were 0.19 and 0.21 at 37 to 40 weeks of age, respectively, and 0.13 and 0.29 at 57 to 60 weeks of age, respectively. N'Dri et al. [28] estimated the heritability of feed efficiency traits in a commercial slow-growing meat-type chicken line and reported that estimates of heritability for FCR and RFI were 0.33 and 0.45, respectively. Although the estimated heritabilities of feed efficiency traits varied between different chicken populations and different studies, estimates for heritability of RFI were generally higher than FCR. In the present study, we estimated the heritability of feed efficiency traits in a broiler population, and estimates for the heritability of FCR and RFI were 0.37 and 0.46, respectively. The results indicated that FCR and RFI have moderately high heritability in broiler populations. Selection for RFI can be expected to be more efficient than selection for FCR in reducing feed cost.

Although the size of the dataset was small in the current study, the standard errors of heritability estimates were low. There are at least three possible reasons for the low standard errors. First, the data were collected from a single farm during the same period; thus, the model was rather simple and only a few parameters needed to be estimated. Second, the dataset had a good structure (appropriate size of half-sibs and full-sibs) with regard to estimating genetic parameters. Third, heritabilities in the current study were estimated based on the genomic relationship matrix built using the 60K SNP chip, which described the relationship between birds more accurately than the pedigree-based relationship. Previous studies [30, 31] reported that genomic information outperforms pedigree in estimating relatedness, and Bérénos et al.[31] reported that the standard errors were lower in analyses based on genomic relatedness.

### Accuracy of genomic prediction for feed conversion ratio and residual feed intake

In the current study, the accuracies of GEBV were evaluated in two ways: correlation-based and model-based theoretical accuracy. The results showed that in both CVF and CVR scenarios, the model-based theoretical accuracy was higher than correlation-based validation accuracy for all traits, except for ADG and ADFI in the CVF scenario. This phenomenon of high model-based theoretical accuracy was also observed in previous studies[11, 32, 33], perhaps because the theoretical accuracy was overestimated due to imperfect linkage disequilibrium between markers and causal genes [34]. However, it was observed that ADG and ADFI in CVF scenario had higher correlation-based validation accuracy than model-based theoretical accuracy. This could be resulted from sampling error due to small data set.

Although the training data set was small, the accuracy of genomic prediction was relatively high in this study. This could be explained as the following. The data were collected from a F_2_ population, which was a newly established line with a small effective population size. In a population with small effective population size, the animals shared large chromosome segments; thus, it is expected that genomic predictions will be more accurate [35, 36].

The accuracy of predicted genomic breeding values for feed efficiency traits has been studied in laying hens. Wolc et al. [7] investigated the efficiency of genomic prediction for RFI in a layer population of a brown-egg pure line. The correlation-based accuracies were 0.13 for the validation birds that were two to three generations apart from the training birds, and 0.38 for the validation birds that were one generation apart from the training birds. In the present study, the accuracy of GEBV for RFI and FCR was assessed in two scenarios. The individuals in the test data set did not have any half/full sib in the training data in the CVF scenario, but in the CVR scenario. Therefore there was a more distant relationship between the test and training birds in the CVF scenario than in the CVR scenario. Correspondingly, the correlation-based validation reliabilities in CVF were 0.089 and 0.309 lower than those in CVR for RFI and FCR, respectively. The model-based theoretical accuracies in CVF were 0.157 and 0.215 lower those in CVR for RFI and FCR, respectively. It was observed that the difference in validation accuracy between the two scenarios (CVF vs. CVR) for RFI was much larger than those for other traits. This could be caused by an underestimation of the validation accuracy for RFI due to sampling errors in the CVF scenario. It was shown that the correlation-based validation accuracy for RFI was lower than that for FCR in the CVF scenario, although RFI had higher heritability, which usually leads to higher accuracy [37].

In the real breeding scheme of poultry, parents are usually ready to be included in training data to predict the breeding value of progenies, but not sibs. In other words, the relationship between test and training birds would be higher than that in CVF and lower than that in CVR. Therefore, it could be expected that the accuracies of genomic prediction in the real scenario of chicken breeding would be between the accuracies of the CVF and CVR scenarios. In line with the higher heritability for RFI than FCR, the trait had a higher accuracy of GEBV than FCR, indicating an advantage of selection for RFI to improve feed efficiency.

The accuracy of genomic prediction for the animals without their own records of FCR and RFI was actually lower than the $\sqrt{h^{2}}$ of the traits. This means that genomic information is less important than individual phenotypic information. However, measuring individual feed intake is not easy and involves individual cages and checking waste feed. However, even for birds with their own records, genomic prediction led to a higher accuracy than conventional genetic evaluation (results not shown). Therefore, genomic prediction provides a good alternative for the genetic improvement of feed efficiency in chickens by selection.

Previous studies have reported strong genetic correlations between FCR and ADG [2, 6, 29]. This indicates that FCR can be efficiently improved by selection for increasing ADG. Because ADG is usually included in the selection index, the extra gain in the improvement of feed efficiency by adding FCR in the selection index could be relatively small. In contrast, RFI is independent of ADG, and the extra increase in the improvement of feed efficiency by adding RFI in the selection index could be relatively large. With higher heritability and more accurate estimates of breeding value for RFI than FCR, selection for RFI in a breeding program will lead to larger genetic improvement for feed efficiency than selection for FCR.

### Impact of improving feed efficiency traits on carcass traits

Carcass traits are highly important in meat-type chicken breeding because they can greatly affect the economic value of the birds. However, carcass traits are difficult to measure, and direct measurements can be obtained only when the individuals are slaughtered. Due to the limitation of phenotypic records, there are very few studies on the relationship between feed efficiency and carcass traits. N'Dri et al. [28] investigated relationships between feed conversion ratio and carcass traits in slow-growing chickens and reported that FCR had a negative phenotypic correlation with leg muscle percentage, but the correlation between FCR and breast muscle percentage was null. In the present study, based on genomic information, the genetic correlation between FCR and LMP was significantly negative. Moreover, the genetic correlations between FCR and BMP and between FCR and EP were also significantly negative. The results suggest that improving FCR could increase LMP, BMP and EP. For RFI, the genetic correlation with all the three carcass traits was also significantly negative. This study was based on a small population, and power analysis was applied to test the differences between the correlation coefficient and zero. For all the carcass traits, the power of the correlations between feed efficiency and carcass traits was acceptable (P>0.85). The results indicate that reducing feed efficiency traits could improve all three carcass traits.

## Conclusions

RFI and FCR are moderately heritable with higher heritability for RFI than FCR. Furthermore, GEBV of RFI was more accurate than FCR. In addition, RFI has a favourable correlation with all three carcass traits and no correlation with ADG. Therefore, RFI could be a good alternative to selection for improving feed efficiency in chicken breeding programs.

## Supporting information

S1 Table Accuracy of genomic predictions for the birds in the test sets of the cross-validation. The accuracies of genomic prediction were evaluated in two ways: the correlation-based accuracy and model-based theoretical accuracy.
(XLSX)
